# Preparation and of PVA-based compositions with embedded silver, copper and zinc oxide nanoparticles and assessment of their antibacterial properties

**DOI:** 10.1186/s12951-020-00702-6

**Published:** 2020-10-21

**Authors:** Jolanta Pulit-Prociak, Anita Staroń, Paweł Staroń, Anna Chmielowiec-Korzeniowska, Agata Drabik, Leszek Tymczyna, Marcin Banach

**Affiliations:** 1 Faculty of Chemical Engineering and Technology, Institute of Chemistry and Inorganic Technology, Cracow University of Technology, Warszawska 24, 31-155 Kraków, Poland; 2 Department of Animal Hygiene and Environmental Hazards, University of Life Sciences, Akademicka 13, 20-950 Lublin, Poland

**Keywords:** Nanosilver, Nanocopper, Zinc oxide nanoparticles, Coating, Antibacterial effect

## Abstract

A series of poly(vinyl alcohol) (PVA) based liquid compositions with addition of zinc oxide, silver and copper nanoparticles has been prepared. The compositions also contained other consistency-forming organic components. The physico-chemical properties of the products have been determined. Their pH and density have been assessed. Also, the size of nanoparticles has been defined with using a dynamic light scattering technique. The compositions were subjected to XRD, FT-IR and microscopic analysis as well. Thanks to the incorporation of both metal oxide and metallic nanoparticles, it was possible to enrich the products with antibacterial properties. Their inhibiting properties in the growth of microorganisms have been confirmed against both Gram-negative and Gram-positive strains such as *E. coli*, *S. aureus* and *P. aeruginosa*. Thanks to the ability for solidification, the compositions may be applied on a bacterially contaminated surface, and after destroying the microorganisms and its solidification, it may be peeled off along with the dead bacterial film.
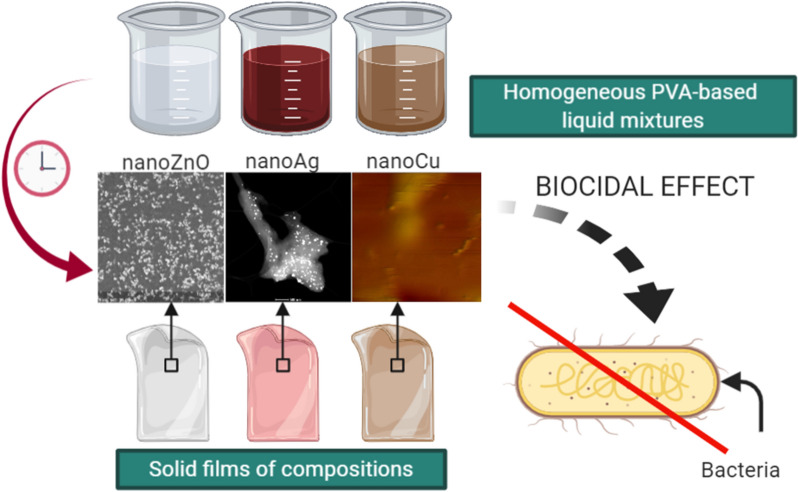

## Highlights

- Homogeneous PVA-based compositions have been prepared.
- Compositions contained among others zinc oxide, silver or copper nanoparticles.
- Thanks to the nanoparticles, the products exhibited biocidal properties.
- Biocidal effect was confirmed against *E. coli*, *S. aureus* and *P. aeruginosa*.
- It is possible to solidify compositions and detach the dead bacterial cells.

## Introduction

Nanotechnology is considered to be one of the most dynamically developing sciences; it allows the improvement of existing products, devices and technological solutions but also allows the creation of new ones by using materials with at least one dimension in the range of 1–100 nm [1]. The growing state of knowledge about nanomaterials and the development of research methods makes it possible to control the size and shape of the obtained nanoparticles, which in turn, gives the materials new, favourable physico-chemical properties. The greatest progress is manifested in areas such as medicine, food technology, electronics, chemical and material engineering, biotechnology and environmental engineering [2]. Nanomaterials are successfully used in the fight against microorganisms. The high effectiveness of nanoparticles against bacteria is of particular importance nowadays because the abuse of antibiotic therapy has resulted in the production of strains resistant to this type of therapy.

The mechanisms of bacterial destruction are varied and depend on the type of nanoparticles. Nanoparticles can penetrate the bacterial wall and form pores on the surface of the membrane, which leads to the formation of free radicals that are able to destroy the cell membrane [3]. In addition, ions formed from nanoparticles can disrupt the production of enzymes and generate reactive oxygen species (ROS), which affect DNA transcription [4].

Among nanomaterials, special attention is paid to nanometric silver. The effectiveness of its biocidal action depends on several factors: the shape, surface and size of the nanosilver particle [5]. Studies have shown that spherical and triangular forms up to 30 nm in size exhibit the highest fighting efficiency against microorganisms [5–11]. The antimicrobial effectiveness of silver nanoparticles is also dependent on the bacterial strain and cell wall structure [12]. Peptidoglycans located on the surface of the cell wall of Gram(+) bacteria are responsible for the proper functioning of the respiratory chain, and as a result of the action of nanosilver, they lose their properties that enable oxygen respiration, which leads to the death of the bacteria [13].

The high antimicrobial effectiveness of copper nanoparticles has also been confirmed [3, 14]. Copper nanoparticles with a diameter not exceeding 100 nm interacts with the surface of the cells of microorganisms, as a result of which, a bipartite copper ion is formed, which enters the cell [15, 16]. In a microbial cell, ions produce reactive oxygen species that lead to lipid peroxidation and protein oxidation [15].

The biocidal effectiveness of nanometric metal oxides (including ZnO, CuO, Fe_2_O_3_, Ag_2_O, MgO, TiO_2_, CeO_2_ and Al_2_O_3_) has also been tested [17–19]. In the case of zinc oxide nanoparticles, the literature does not explicitly state the effectiveness of their action against Gram-positive (G(+)) and Gram-negative (G(−)) bacteria [20, 21]. Tayel et al. [22] examined the antibacterial properties of zinc nanooxide against nine bacterial strains and observed that Gram-positive bacteria are more sensitive to its effects than Gram-negative bacteria. Reddy et al. had similar observations [23]. They showed that to inhibit the growth of Gram-negative bacterial cells, three times higher concentration of zinc oxide nanoparticles is necessary than in the case of Gram-positive bacteria. In turn, different observations were described by Pasquet and Applerot and colleagues [20, 24]. According to their research, zinc oxide nanoparticles show a stronger fighting effect against bacteria built of the outer cell membrane. Therefore, the proposed mechanisms describing the operation of nanoZnO are also different. It is assumed that in the case of Gram-positive bacteria, zinc oxide adheres to peptidoglycan or the double lipid layer is characterised by high sensitivity to ROS produced as a result of ZnO activity [24].

The undoubted advantage of using metallic nanoparticles in the fight against microorganisms is the non-specific bacterial toxicity of these nanoparticles. According to this mechanism, nanoparticles do not bind to a specific receptor in a bacterial cell, thanks to which resistance of these bacteria does not develop [25].

The article presents research on the antibacterial activity of poly(vinyl alcohol) (PVA)-based compositions containing silver, copper and zinc oxide nanoparticles. It is intended that the composition may be applied in a liquid form on a surface that is contaminated with microbial cells. The great feature of the products is that after a few hours, it is completely solidified. Thus, after application and solidification, the composition may be detached with dead biological film. The minimal inhibiting concentration (MIC) and bacterial cell reduction against both Gram-positive and Gram-negative bacteria (*Staphylococcus aureus*, *Escherichia coli* and *Pseudomonas aeruginosa*) was determined.

## Materials and methods

### Materials

The following compounds were used in this study: poly(vinyl alcohol) (M = 72 kg/mol, ≥ 99.0%), sucrose (≥ 99.5%), 2-hydroxyethyl cellulose (M = 90 kg/mol, p.p.a.), guar gum (p.p.a.), gelatine (p.p.a.), chitosan (M = 100–300 kg/mol, high purity), glycerol (d = 1.26 × 10^3^ kg/m^3^, p.p.a.), casein (p.p.a.), zinc nitrate (≥ 99.9%), silver nitrate (≥ 99.0%), copper chloride (99.0%), tannic acid (ACS reagent), sodium hydroxide (≥ 98.0%) and acetic acid (≥ 99.0%). All compounds were obtained from Sigma-Aldrich. Test microorganism strains included in the study were as follows: *Escherichia coli*, *Staphylococcus aureus* and *Pseudomonas aeruginosa*. They were purchased from MicroBioLogics, Inc., (St. Cloud, USA). Mueller–Hinton broth, Tryptic Soy Agar (TSA) and Ringer solution were purchased from BTL Polska Sp. z o. o., Poland. All aqueous solutions were prepared using deionised water (Polwater, 0.18 µS).

### Methods

#### Preparation of PVA-based coatings with nanoparticles

In this study, a series of PVA-based compositions with zinc oxide, silver and copper nanoparticles were prepared. The base formulation was the same for all types of products. The affinity of the prepared products with nanoparticles to microbiological biofilm should be enhanced. Thus, the specific compounds thanks to which the “hook effect” is achieved have been used. The organic matrix of all compositions consisted of sucrose, hydroxyethyl cellulose, guar gum, gelatine, chitosan, glycerol and casein. The adhesion of microorganisms to any surface is possible thanks to extracellular polymers which are secreted out. This group is consisted of lipopolysaccharides and proteins. Thanks to the fact that the products contain compounds such as casein (protein), sucrose (carbohydrate) or chitosan (polysaccharide), their adhesion to the surface will be increased. The adhesion of microbial cells to any surface should be considered as a multistep process. It depends on hydrodynamic forces, gravity, thermodynamic (Brownian motion) forces and van der Waals forces. The van der Waals forces make it possible to attract the cells to the material surface in an efficient way. If the distance between microbial cells and the surface is less than 1.5 nm, then a hydrogen bonding and covalent carbon–carbon bonds are produced. The better adhesion of applied compositions is ensured by a chemical interaction of substances such as casein, sucrose, or chitosan with extracellular polymers that are secreted out by biofilm [26–28]. In the beginning, the aqueous solutions of these components were prepared. Chitosan was dissolved in acetic acid solution (1.5% v/v). Since casein is freely soluble in an alkaline environment, it was dissolved in NaOH solution at different concentrations. In order to obtain compositions with zinc oxide, its previously prepared solid phase was added directly to the dispersed composition. Zinc oxide nanoparticles were prepared according to a previously developed method [29]. Both silver and copper nanoparticles were formed in situ in the course of the compositions preparation. For this purpose, the aqueous solution of silver or copper ions source was introduced to the PVA-based mixture. Silver nitrate solution and copper chloride solution served as ion suppliers. Tannic acid aqueous solution was used as both a reducing and stabilising agent. Since the chemical reduction using tannic acid is promoted by an alkaline environment, it was provided by the addition of a casein alkaline solution. Antibacterial compositions were prepared using a mechanical stirrer and elevated temperature. First, PVA was dissolved in deionised water. After that, the rest of the reagents were added one by one with the order provided in Table 1. The concentrations and volumes of all reagent solutions added made it possible to obtain compositions with the final concentrations of all ingredients that are presented in Table 1. The reference product did not contain the nanoparticles. Table 1 also provides other process parameters. In order to ensure good dispersion of all components, the mixture was stirred for another 10 min. Each process led to the obtaining of 40.0 g of antibacterial composition.

**Table 1:** Concentration values of components in individual compositions, process parameters and properties of the products

Component	Concentration of the component in the final product										
	Compositions with nanoZnO			Compositions with nanoAg				Compositions with nanoCu			Ref.
	K1	K2	K3	K4	K5	K6	K7	K8	K9	K10	
PVA, %	7.0										
Sucrose, %	0.12										
Hydroxyethyl cellulose, %	0.10	0.10	0.01	0.10	0.10	0.01	0.01	0.10	0.10	0.055	0.10
Guar gum, %	0.01	0.05	0.01	0.05	0.05	0.05	0.05	0.05	0.05	0.05	0.05
Gelatine, %	0.05	0.5	0.5	0.1	0.5	0.3	0.5	0.5	0.5	0.1	0.3
Chitosan, %	0.01										
Glycerol, %	14.0										
Casein, %	0.4										
ZnO, %	3.0	5.0	5.0	–	–	–	–	–	–	–	0
Silver nitrate + tannic acid → nanoAg, ppm	–	–	–	1000	500	200	200	–	–	–	0
Copper chloride + tannic acid → nanoCu, ppm	–	–	–	–	–	–	–	1000	200	200	0
	Process parameters										
C_NaOH_, mol/l	0.5	0.3	0.3	0.3	0.3	0.3	0.9	0.5	0.3	0.3	0.5
T, °C	80	80	60	80	80	60	20	60	80	60	60
	Properties of the products										
pH	7.12	6.81	6.92	5.50	5.44	6.29	7.38	5.38	5.17	5.12	6.12
Density, g/ml	1.111	2.997	1.410	2.903	2.906	1.125	1.105	2.925	2.939	2.915	2.925
Size, nm (DLS)	720	1215	580	1464	407	1544	679	762	186	1489	-
Size, nm (AFM)	300–600	300–400	350–600	250	200	250	200	400	300	350	-

The physico-chemical properties of the liquid form of obtained compositions have been analysed. The pH values were checked using an Elmetron pH electrode. The density of compositions was assessed by the weighting method. By using a dynamic light scattering technique (DLS), it was possible to determine both the size and electrokinetic potential of the nanoparticles. The measurement was performed by Zetasizer Nano, Malvern. Before the measurement, it was necessary to prepare the samples in a specific way. Aqueous suspensions of the compositions at a concentration of 50 ppm have been prepared. In order to suspend the compositions in the dispersing agent, the suspensions were homogenised for 1 min using an ultrasonic homogeniser (Hielscher UP50H). Compositions with silver nanoparticles were tested by a spectrophotometric method (UV 1800, Rayleigh). For this purpose, compositions have been diluted with deionised water to the concentration of silver equal to 50 ppm. In order to minimise the influence of the organic matrix, the spectra of reference sample was subtracted from spectra of the compositions. The products are intended to be used in a liquid form applied on a microbiologically contaminated surface and, after its solidification, to be peeled off along with the dead biological content. In order to perform analyses that are suitable for solid samples, the compositions have been solidified. For this purpose, 2 ml of liquid products have been put on a Petri dish and left for their solidification. As prepared, the solid films were subjected to further analysis. The crystal structure was assessed in the course of X-Ray Diffractometry (XRD) using X’Pert PW 1752/00, Philips. In order to detect the characteristic functional groups contained in the products, the solid films were subjected to Infrared Spectroscopy with Fourier Transformation (FTIR). The samples were scanned over the range of 400–4000 cm^−1^ in a Nicolet 380 spectrophotometer (Thermo Fisher). The distribution of nanoparticles in the solid products was determined by both scanning electron microscopy (SEM) with energy dispersive X-ray spectrometry (EDX) and atomic force microscopy (AFM). The studies were carried out using a 1430 VP microscope (LEO Electron Microscopy) and MultiMode microscope with NanoScope IIIa and Quadrex controller (Veeco Digital Instrument).

In order to compare the effectiveness of prepared compositions, the antibacterial properties of pure nanoparticles have also been investigated. Their physico-chemical properties have also been checked. The powder of ZnO was assessed by XRD, FTIR and SEM techniques. Suspensions of nanoAg and nanoCu at a concentration of 1000 ppm have been prepared based on a reduction reaction by mixing 36 ml of metal ion source aqueous solution with 4 ml of tannic acid aqueous solution. The concentration of silver nitrate and copper (II) chloride solutions were equal to 0.01 mol/l and 0.0315 mol/l, respectively. The concentrations of tannic acid solutions were equal to 0.0185 mol/l (in the case of obtaining nanoAg) and 0.0315 mol/l (in the case of obtaining nanoCu). These amounts ensured the molar volume of reducing agent to metallic ions equal to 0.2:1.0. After mixing the source of metallic ions with a reducing-stabilising agent, the pH of the mixture was adjusted up to 8 using NaOH aqueous solution at a concentration of 0.1 mol/l. These suspensions were also analysed by instrumental techniques. The sizes of the nanoparticles were checked by DLS technique. The UV–Vis spectroscopy was applied to identify silver nanoparticles. The pure nanoparticles were also tested in microbiological studies. For this purpose, both silver and copper aqueous suspensions were taken at a concentration of 1000 ppm. Zinc oxide powder was suspended in water so that the final concentration of ZnO was equal to 5%. In order to homogenise the product, the suspension was pontificated for 1 min.

#### Analysis of antibacterial properties of obtained compositions

In the study, the minimal inhibiting concentration (MIC) and the minimal biocidal concentration (MBC) were determined. The evaluation of the biocidal effect of the tested materials was carried out by means of both serial two-fold dilution method and a suspension method. The studies were performed against indicator microorganisms, including Gram-positive bacteria (*Staphylococcus aureus*) and Gram-negative bacteria (*Escherichia coli* and *Pseudomonas aeruginosa*).

##### Preparation of tested materials

5 ml of Mueller–Hinton broth (MH) was poured into ten sterile tubes. There was 5 ml of the tested composition introduced into the first tube (1:1 dilution). From the mixture thus obtained, 5 ml was transferred to another tube with broth. In this way, further two-fold dilutions were obtained (1:2, 1:4, 1:8, 1:16, 1:32, 1:64, 1:128, 1:256 and 1:512).

##### Inoculum preparation

Inoculum was prepared from a 24-h bacterial culture (on TSA media). For this purpose, 3–5 cells were harvested and transferred to 5 ml MH broth. The whole was incubated for 2–3 h at 35 °C. After this time, the inoculum density was set at 0.5 McF by using a densimeter. This value corresponds to a density of 1–2 × 10^8^ cfu/ml. Inoculum with higher turbidity was adjusted with MH broth.

##### Determination of MIC and (MBC)

There was 0.1 ml of inoculum introduced into the prepared tubes with the medium and the tested materials. The control was a tube with MH broth (10 ml) without the addition of the preparation (zero test). The results were assessed after 24 h of incubation at 37 °C. The MIC concentration was the concentration of the tested material that completely inhibited the growth of microorganisms. The assessment consisted in measuring the turbidity of the medium after incubation. In order to determine the MIC, the bacterial culture considered negative was used to perform inoculation on TSA medium without the addition of nanoparticles. Samples were incubated for 24 h at 37 °C. The concentration of the material causing complete inhibition of bacterial growth was defined as MBC (macroscopic evaluation).

##### Determination of bacterial cell reduction

Inoculates of 0.5 McFarland density and their dilutions Z^−1^, Z^−2^ and Z^−3^ were prepared from fresh (24 h) cultures of microorganisms. Then, to four tubes containing 9 ml of each of the compositions K1, K5, K7 and K9, 1 ml of suspension from dilution Z-2 was added. The Z-3 suspension in Ringer's solution was a control sample. The study was performed according to Polish Standards PN-EN 1040 [30] and PN-EN 1275 [31]. In accordance with them, the preparation can be considered as meeting the requirements for the basic bactericidal and fungicidal activity if, under the test conditions, within 60 min, it causes a 10^5^-fold reduction of viable bacteria and a 10^4^-fold reduction of viable fungi. Based on the standards, the contact time should be equal to 5 min and 1 h. After that time, 0.1 ml of suspension from each tube was inoculated onto agar plates (TSA for *S. aureus*, McConkey for *E. coli* and enriched agar for *P. aeruginosa*). The plates were incubated for 24 h at 37 °C. After this time, the grown colonies were counted. On their basis, the logarithmic reduction ratio was calculated.

## Results and discussion

### PVA-based coatings with nanoparticles

Ten PVA-based compositions have been obtained. The products differed in colour. Compositions with zinc oxide nanoparticles were white. The presence of both silver and copper nanoparticles led to the formation of reddish or brownish products.

**Fig. 1:**
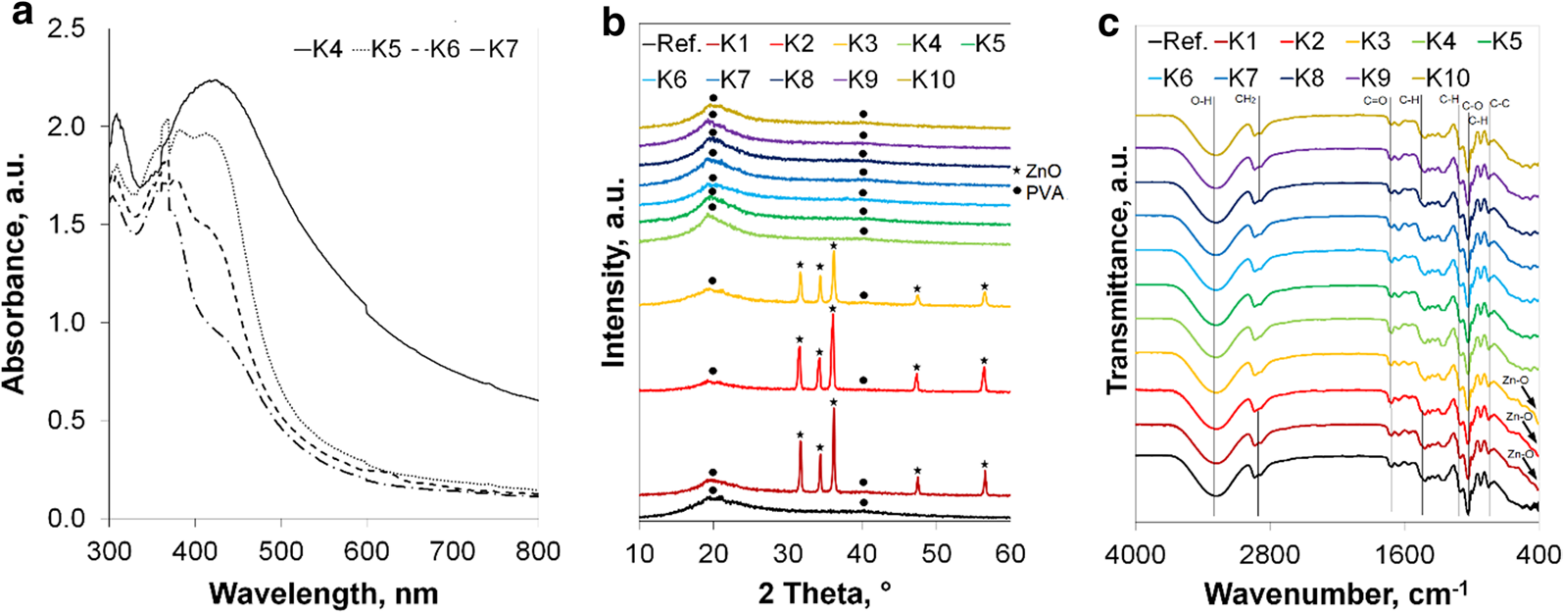
**a** UV–Vis spectra of liquid compositions; **b** XRD diffractogram of all compositions in solid phase and **c** FTIR spectra of all compositions in the solid phase

The pH values of prepared compositions are provided in Table 1. The pH was in the ranges of 5.12–5.38 (compositions with nanoCu); 5.44–7.38 (compositions with nanoAg) and 6.81–7.12 (compositions with nanoZnO). It was obvious that a higher concentration of sodium hydroxide solution, which was used to dissolve casein, caused a higher pH of the product. Table 1 presents the results of the density of the measured compositions. One may conclude that the density is affected by both hydroxyethyl cellulose and guar gum content. Gelatine does not seem to influence this parameter. These observations are in-line with other researchers' studies. Both hydroxyethyl cellulose and guar gum are able to rapidly increase the density of consumer products. They are applied in order to moderate the density and viscosity of materials [32–35]. The measured size values of zinc oxide, silver and copper nanoparticles are provided in Table 1. The interpretation of these results must be critical. Since the DLS technique is based on the Mie theory, one should be aware that it is assumed that particles are spherical. It may be especially applied in the interpretation of zinc oxide particles. Flower-like particles may be approximated to spherical shape, and thus, their size seems to be bigger. The influence of the organic matrix poses another problem. Even though the refractive index, which is characteristic for analysed particles, has been set during the measurements, there were still macromolecules that could affect the values provided by the apparatus [36]. This may be related to the multiple scattering effect, which can lead to misleading results. Thus, microscopic techniques are better for assessing the size of nanoparticles in such products. Figure 1a presents the results of spectrophotometric analysis. Thanks to the surface plasmon resonance, silver nanoparticles have a UV–Vis absorption maximum within the range of 400 and 500 nm [17, 37]. The spectra of compositions with silver nanoparticles after subtracting the background originating from the reference sample exhibit characteristic peaks. Unfortunately, despite efforts to avoid the influence of organic matter, due to the organic compounds which are were still present, an increase in background is observed that is caused by the absorption of all other unmarked components of the sample. However, this fact does not interfere with the successful identification of nanosilver in the tested compositions. Diffractograms of solid phase of obtained compositions are presented in Fig. 1b. It is clearly seen that zinc oxide nanoparticles contained in K1–K3 compositions may be identified. This was confirmed by reflections at 31.6°, 34.4°, 36.1°, 47.6° and 56.6° of 2θ angle [38, 39]. This pattern is in line with the lattice planes (100), (002), (101), (102) and (110) respectively [40]. The plots of compositions with copper and silver nanoparticles did not differ from the plot of the reference sample. This technique was not adequate to reveal silver and copper nanoparticles presence in the products. This is due to the fact that the concentration of both silver and copper nanoparticles in the tested products was too low. The concentration of nanoparticles included in K4-K10 samples was from 50 to 250 times lower than in compositions with zinc oxide nanoparticles. The reflections originating from organic matter covered the peaks obtained for silver and copper nanoparticles. The lack of other clear reflections in the X-Ray results makes it seem that the compositions were loaded with amorphous or semi-crystalline substances. The semi-crystalline structure of PVA was confirmed by reflections at 19.5° of 2θ angle [41]. The incorporation of neither silver nor copper nor zinc oxide nanoparticles did not worsen the intensity of these major peaks, which was the desired effect. The amorphous phase of PVA may be identified by the reflections at around 40°. The intensity of the major amorphous reflection may be enhanced by the presence of the rest of organic matter. The applied substances also exhibit a Bragg’s reflection at around 20° of 2θ angle [42–47]. In order to assess the bonds in the prepared compositions, FTIR spectra of the solid form of the obtained products have been recorded in the range 400–4000 cm^−1^. FTIR spectra of all solid compositions are presented in Fig. 1c. The identification of ZnO in K1–K3 compositions may be done based on peaks observed at 410 cm^−1^. These peaks correspond to Zn–O symmetric bending vibrations. The major peaks at 3300 cm^−1^ come from O–H stretching vibrations. This groups originate from water molecules, which are adsorbed on the compositions' surfaces [48, 49]. The peaks observed at 2930 cm^−1^ may be due to CH_2_ stretching vibrations [50]. Peaks at 1710 and 1415 cm^−1^ may be assigned to C=O carbonyl stretching vibrations and C–H bending vibrations [51] of the CH_2_ group, respectively. The presence of C–H deformation vibrations, C–O stretching and C–C stretching vibrations is confirmed by peaks at 1324, 1097 and 840 cm^−1^ [49], respectively. Peaks at 1030 cm^−1^ may originate from C–H deformation vibrations of the aromatic groups [52]. These peaks confirm the presence of organic matter in the obtained products. The appearance of spectra of compositions with nanoparticles does not differ from the reference product, which means that the organic matrixes were not changed due to the incorporation of inorganic nanoparticles.

**Fig. 2:**
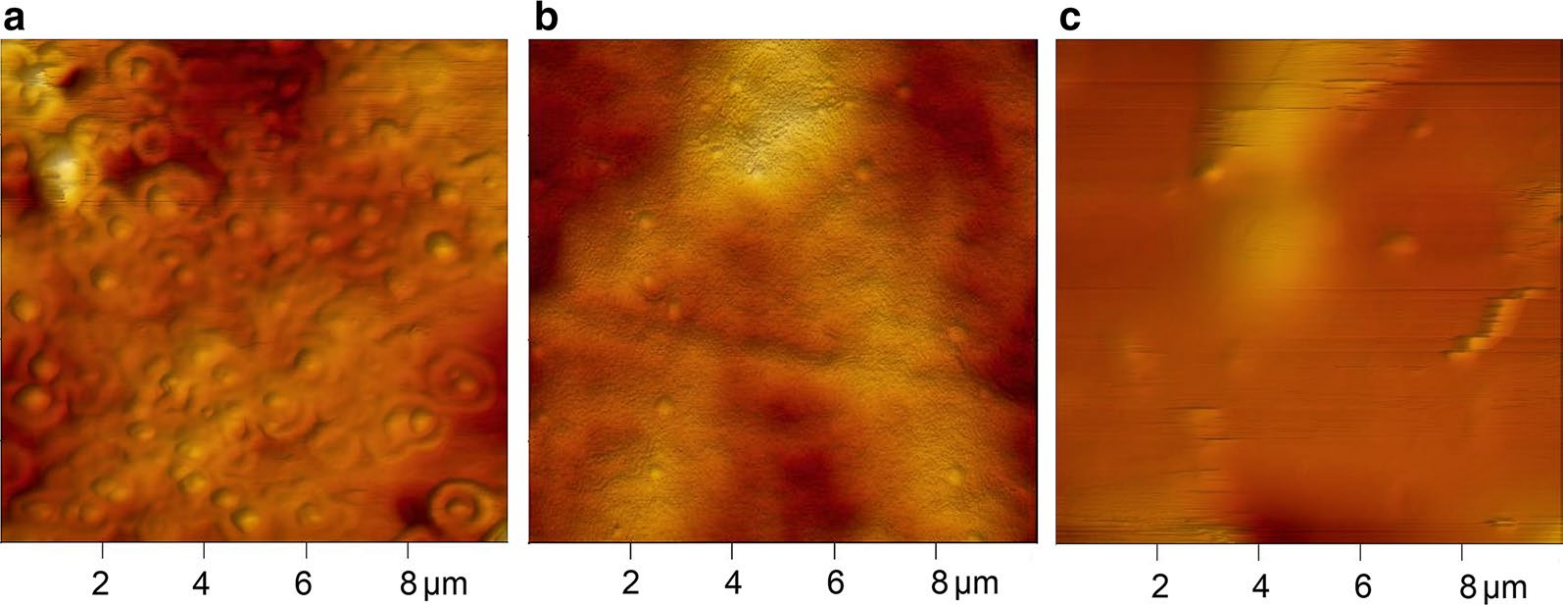
AFM microphotographs of compositions **a** K1; **b** K5 and **c** K9

Figure 2 presents the exemplary results of microscopic analysis. AFM microphotographs show the shape and size of nanoparticles. All of them were spherical and well dispersed in the organic matrix. The size of the nanoparticles differed from the results of DLS analysis. Based on the AFM microphotographs, one may observe that the size of ZnO nanoparticles in the K1 composition was in the range of 300–600 nm. Silver nanoparticles in the K5 composition did not exceed 200 nm, and the size of copper nanoparticles in the K9 composition was equal to 300 nm. The size of nanoparticles contained in the rest of compositions are provided in Table 1.

**Fig. 3:**
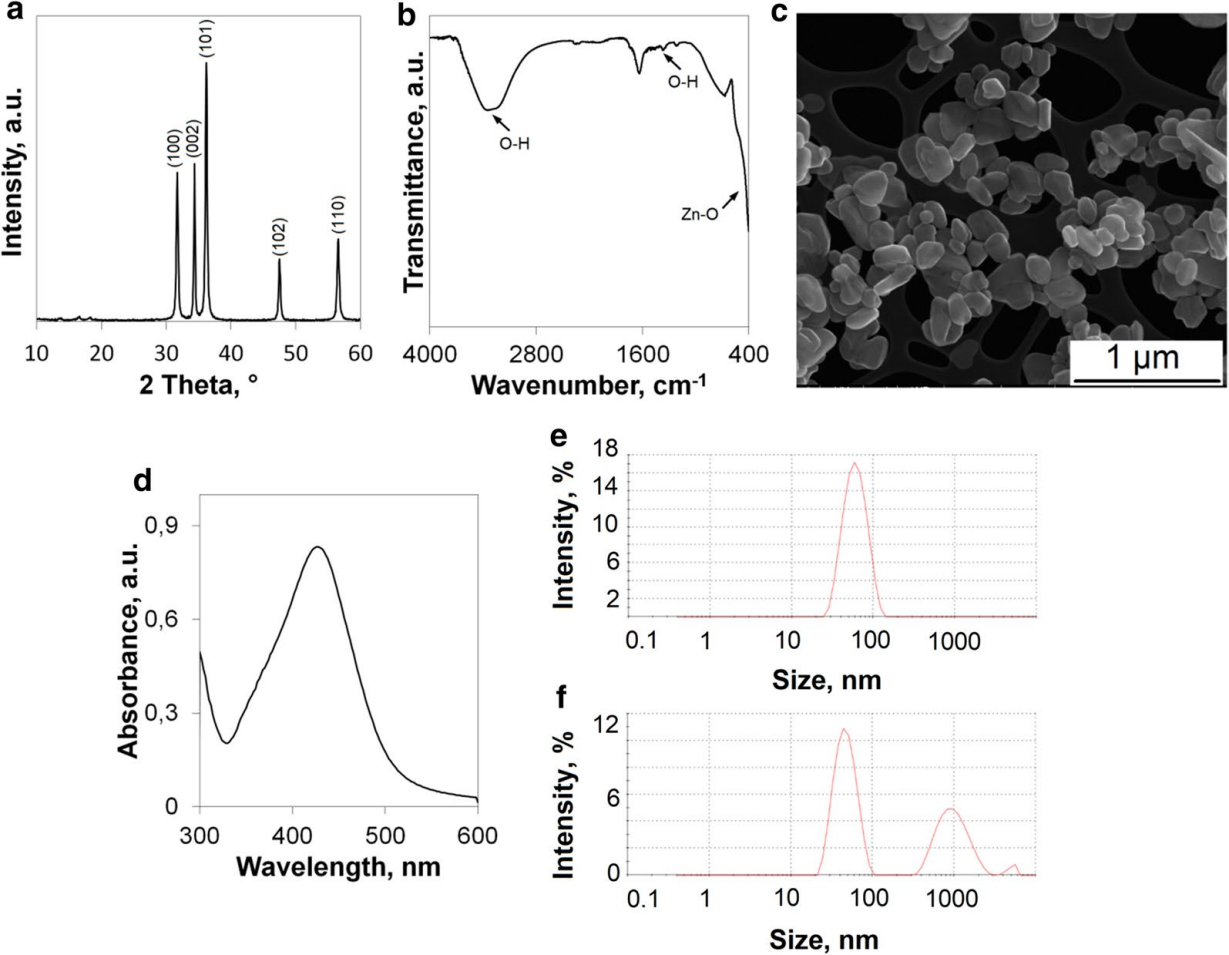
Results of analysis of physico-chemical properties of pure nanoparticles **a** XRD of nanoZnO; **b** FTIR of nanoZnO; **c** SEM microphotographs of nanoZnO; **d** UV–Vis spectra of nanoAg; **e** DLS histogram of nanoAg and **f** DLS histogram of nanoCu

Figure 3 presents the results of analysis of physico-chemical properties of pure nanoparticles. XRD diffractograms of ZnO nanoparticles are shown in Fig. 3a. The characteristic reflections are clearly visible. The peaks at 31.6°, 34.4°, 36.1°, 47.6° and 56.6° of 2θ angle confirm the presence of zinc oxide [38, 39]. The diffractogram matches with the lattice planes (100), (002), (101), (102) and (110) respectively [37]. FTIR spectra of pure zinc oxide is presented in Fig. 3b. One may expect the band in the region below 1000 cm^−1^, which proves the bonds between metal and oxygen [53]. The band occurring between 400 and 580 cm^1^ confirms the stretching vibration of Zn–O. The peak at around 1400 cm^−1^ may be assigned to the H–O–H bending vibration, which occurs due to the adsorbed moisture on the zinc oxide surface. The presence of water may be also confirmed by a wide band between 3000 and 3650 cm^−1^. The size of zinc oxide nanoparticles may be assessed based on SEM microphotography (Fig. 3c). One may conclude that the smallest dimensions of particles range from 80 to 300 nm. Their shape is spherical or the particles are like elongated balls. The edges of particles are clearly seen, which means that they are well dispersed. Figure 3d shows the UV–Vis spectra of aqueous suspension of silver nanoparticles diluted to 50 ppm. As expected, the major peak occurs between 400 and 500 nm, and its maximum absorbance is at λ = 434 nm. That confirms the obtaining of pure silver nanoparticles [17, 37]. The results of both nanoAg and nanoCu size measurements are provided in Fig. 3e, f. There is only one dominant size of silver nanoparticles that is manifested by a thin peak whose maximum occurs at 62 nm. In the case of copper nanoparticles, one may observe three peaks. The first major peak is at 48 nm (60.2%). There are also some bigger agglomerates of nanoparticles, and their presence is confirmed by the second peak at 1010 nm (38.1%). Only 1.7% of nanoparticles form agglomerates with a size of 5012 nm.

### Antibacterial properties of obtained coatings

**Fig. 4:**
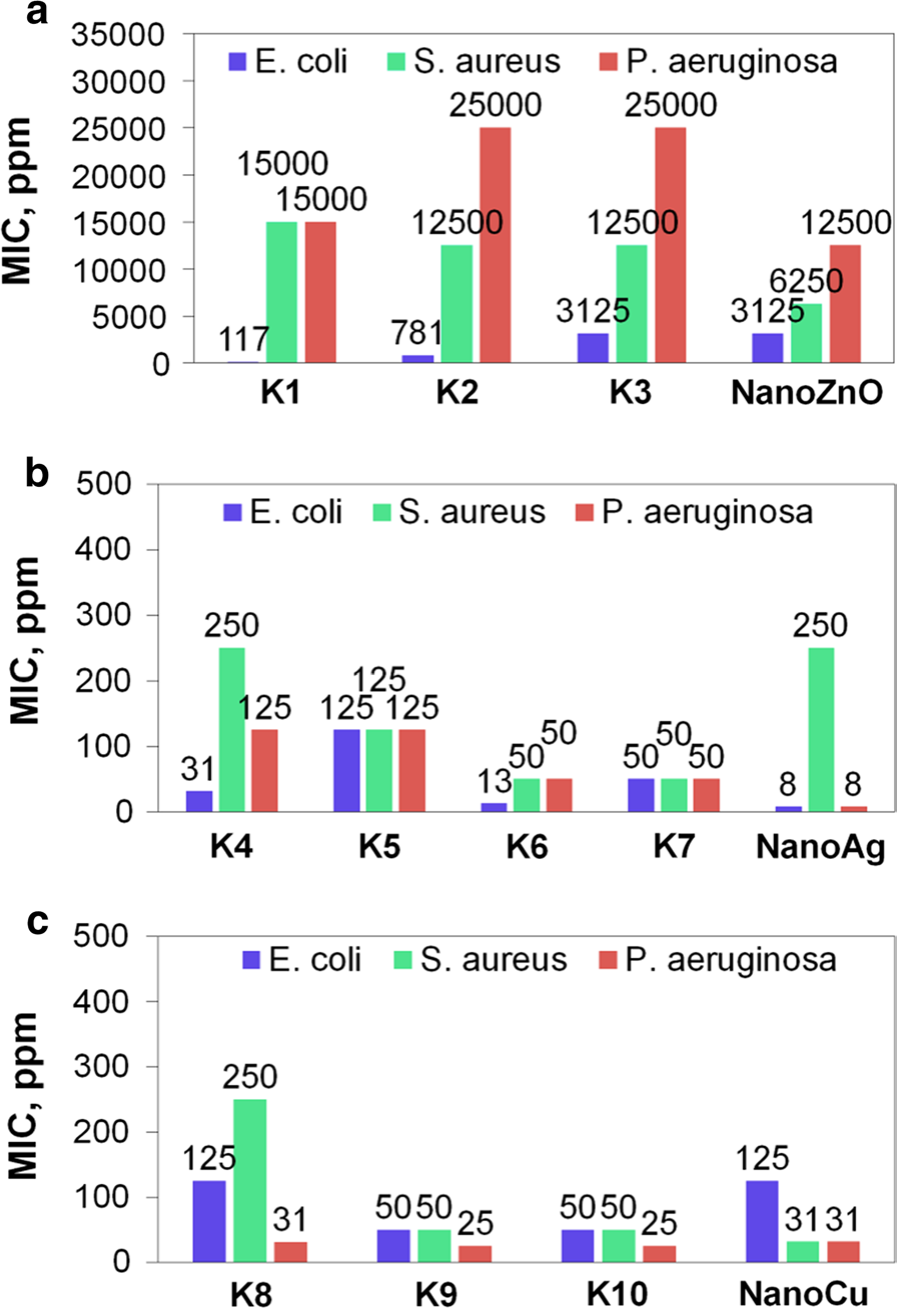
**a**–**c** Results of MIC analysis against bacteria strains **a** compositions with nanoZnO; **b** compositions with nanoAg and **c** compositions with nanoCu)

The analysis of anti-microbial properties was conducted against three bacteria strains. Two of them were Gram-negative bacteria (*E. coli* and *P. aeruginosa*), and the *S. aureus* strain was a Gram-positive bacteria. Figure 4 presents the MIC results determined for all tested products (compositions, aqueous suspensions of pure nanomaterials and the reference sample). For easier interpretation of the results, the minimum inhibitory concentration for zinc oxide is expressed in *ppm* unit. Such a notation allows the data to be compared. The reference product did not exhibit any inhibiting properties in the growth of the tested bacteria. It was a great medium for development of the strains. In fact, the reference sample consisted of organic matter, and it was a reach source of carbon for the microorganisms [54–56]. Despite the fact that the matrix of the compositions with nanoparticles was the same as in the reference product, their antimicrobial properties may be observed. Analysis of the influence on the *E. coli* strain may conclude that the best inhibiting properties were provided by silver nanoparticles in both forms (compositions and as a pure agent). The action of copper nanoparticles in both forms was a little bit worse; however, it remained at a similar level. Zinc oxide nanoparticles in pure form and incorporated into the compositions structure exhibited the worst biocidal properties against the *E. coli* strain. This comparison is based on the MIC values expressed in the unified unit. Zinc oxide nanoparticles, in general, exhibited the weakest antibacterial activity. This was also confirmed in the case of both *S. aureus* and *P. aeruginosa*. Copper nanoparticles in both forms exhibited the strongest inhibiting properties against those bacteria. Silver had a moderated influence on the development of *S. aureus* and *P. aeruginosa*. Comparison of different forms of nanoparticles showed other dependencies. Not all pure aqueous suspensions of the tested nanoparticles exhibited better activity than nanoparticles included in the structure of the tested compositions. One might have expected that pure nanoparticles have stronger inhibiting properties in the development of bacterial strains. However, these studies confirmed that even though the active agent was incorporated in the organic matrix, it also had the ability to destroy the microorganisms. In the case of *E. coli* (G(−)), only pure nanoAg exhibited better inhibiting properties. In order to inhibit the growth of this strain, the compositions with nanoAg at a concentration of 8 ppm had to have been applied. In general, both compositions with nanoZnO and nanoCu had a better antibacterial activity than the pure nanoparticles. The opposite phenomena may be observed in the case of the Gram-positive bacteria, *S. aureus*. Neither ZnO nor Cu nanoparticles contained in the compositions exhibited as strong an inhibiting action as in the case of the pure nanoparticles. Only compositions with nanoAg (K5, K6 and K7) were more effective agents compared to the pure nanoAg. These results may be related to the structure of the cell wall of Gram-positive bacteria. The murein molecules, which consist of long polysaccharide chains cross-linked by peptide bridges that are located in such bacteria, may be a more effective barrier against attacking agents. ZnO and Cu nanoparticles eluted from compositions are probably still covered by a huge molecules of stabilising organic components, and thus, they are not able to interrupt the wall in the same manner as pure nanoparticles. Only silver, which is a quite strong antibacterial agent, manages with that despite being surrounded by other molecules. However, one may observe that the action of silver against Gram-negative bacteria, G(−), is still better than in the case of Gram-positive bacteria, G(+). That also confirms the huge influence of the structure of the cell wall on the activity of the biocidal agent. The Gram-negative bacterial cell wall differs from Gram-positive bacterial cell wall structure. The thickness of Gram-negative bacteria cell wall is about 30 nm while the thickness of Gram-positive bacteria is around 3–4 nm [57]. The cell wall of G(−) bacteria consists of a cytoplasmic membrane, a thin peptidoglycan layer and the external membrane that contains lipopolysaccharide. Contrary to G(+) bacteria, the cytoplasmic membrane is further away from the outer membrane. Also, the network of peptidoglycan chains is more loosely packed. This is the reason for more free penetration of the irritant into the cell [58].

The action of pure ZnO and Cu nanoparticles was better in the case of *P. aeruginosa* (G(−)) bacteria. These results confirm that as opposed to the predictions, not only pure nanoparticles exhibit more effective inhibiting properties in the development of different bacteria strains. The particles incorporated in the structure of PVA-based compositions also have the ability to move and be released from the product and act as effective antibacterial agent. Seong et al. [59] suggested that the mechanism of antibacterial action of nanoparticles may be explained by their direct contact with cells. Nanoparticles may plug the spaces in the microorganism membrane. That causes physical changes in in the membrane which leads to its damage. As a consequence, a leakage of cell content occurs and the bacteria cell is not able to stay alive anymore. What is more, the electric charge of cell membrane is another issue of matter. Negative electric charge which origins from the presence of carboxylic, phosphate and amin groups attracts the positive charged nanoparticles [60].

Another issue which should be discussed is the size of nanoparticles. In the case of Gram(−) bacteria this factor is extremely important when assessing the antibacterial activity. It was demonstrated that the strongest antibacterial effect on Gram(−) bacteria was performed by silver, copper and zinc oxide nanoparticles, respectively. However, here the size of nanoparticles should be considered. In fact, the strongest antibacterial effect was achieved in case of using compositions containing the smallest nanoparticles and the activity was weaker when the particles were greater. This phenomenon may be explained by the structure of Gram(−) membrane which is much more packed. This fact results in that the smallest nanoparticles are most likely to penetrate the cell membrane.

In analysing the process parameters in obtaining the tested compositions, one may note that in the case of compositions with silver nanoparticles, the best results were achieved when the concentration of hydroxyethyl cellulose was the lowest. This resulted in lower density of the product. That means that the particles were more moveable when the density was lower. The density was also the deciding factor when using compositions with zinc oxide nanoparticles. What is more, the temperature of processing was another important factor. The higher temperature led to the obtaining of more active compositions. This may be related with the higher degree of nanoparticles dispersion, which is achieved at a higher temperature of the process. This observation concerned all types of compositions. In the case of compositions with copper nanoparticles, it may be assumed that the size of particles influenced the inhibiting activity. The smaller the nanoparticles were, the more effective action was achieved.

**Fig. 5:**
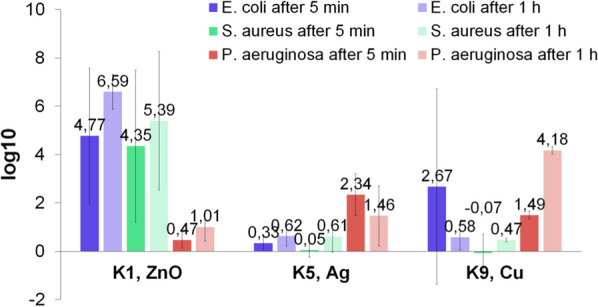
Logarithmic reductions of tested strains after 5 and 60 min (n = 4)

Figure 5 presents the results of logarithmic reductions of tested strains after 5 and 60 min of contact with several chosen compositions. It may be seen that in the case of compositions with nanoZnO, the logarithmic reduction of all strains was higher after a longer time of contact. The biocidal effect against Gram-negative bacteria was also greater after a longer time when using compositions with nanoAg and nanoCu. In some cases, (K5 vs *P. aeruginosa* and K9 vs *E. coli*), the inhibiting action was weaker after a longer time of contact. This may be due to the fact that after 1 h of exposure, bacteria started to consume the tested materials. These compositions had the highest content of hydroxyethyl cellulose, guar gum and gelatine which may have served as an energy source.

Silver nanoparticles have been confirmed to exhibit satisfying anti-microbial properties in studies of other researchers. Panpaliya et al. [61] conducted an analysis of biocidal activity of nanosilver against strains such as *Streptococcus mutans*, *Streptococcus oralis*, *Candida albicans*, *Lactobacillus acidophilus* and *Lactobacillus fermentum*, which are common dental pathogens. In their studies, they used both nanoAg nanoparticles at size of 30–50 nm and Chlorhexidine Gluconate 2% v/w, which is used as commercial biocidal product. It has been found that nanoAg suspension is a five times more effective destroying agent than the commercial formulation. The lowest values of MIC for silver nanoparticles were equal to 2.81 mg/dm^3^ (against *Candida albicans*) and 15 mg/dm^3^ (against *Lactobacillus acidophilus*). The medium value of 45 mg/kg was achieved against *Streptococcus oralis*. Higher MIC values were obtained against *Streptococcus mutans* (60 mg/dm^3^) and *Lactobacillus fermentum* (90 mg/dm^3^). Concerning the fact that the PVA-based compositions contained also organic matrix, the obtained values of MIC are very satisfying.

Similar studies were performed by Petrus et al. [62]. The researchers applied commercially available aqueous suspension of colloidal silver at a concentration of 100 mg/dm^3^ with particles sized from 1 to 100 nm. The activity of nanosilver was tested against the *Escherichia coli*, *Listeria monocytogenes*, *Salmonella enterica* s*erovar* Typhi, *Vibrio cholerae*, *Vibrio parahaemolyticus*, *Bacillus cereus* and *Staphylococcus aureus* strains. These microorganisms are known to be common foodborne pathogens. It turned out that nanosilver is an effective agent against both G(+) and G(−) bacteria. The values of MIC varied depending on the specific strain. The lowest inhibiting concentration was equal to 7.71 mg/dm^3^ (against *Vibrio cholerae* witch is G(−) bacteria). The highest MIC was achieved against *Listeria monocytogenes* (24.58 mg/dm^3^), which is also a G(−) bacteria. Despite the fact that from statistical point of view there is no difference between the activity against different type of bacteria (G(+) and G(−)), a slightly more effective activity may be observed against Gram-negative bacteria. This is in-line with the results of experiments provided in this paper. Kędziora et al. [63] presented very interesting studies on defining anti-microbial properties of nanocomposites consisting of partially reduced graphene with incorporated nanosilver with nanoparticles of 5–20 nm. The bacterial standard strains, such as *Staphylococcus aureus*, *Escherichia coli* and *Klebsiella pneumoniae* (as well as their clinical isolates), were treated with aqueous suspension of the nanocomposite at varied concentrations (0.25–512 mg/dm^3^). These bacterial strains were chosen due to the fact that they very often colonise wounds, including iatrogenic and hospital infections. Similar to the results presented in this paper, the authors concluded that Gram-negative bacteria are more sensitive to the activity of nanosilver compared to Gram-positive bacteria. The bacteriostatic concentration values against all bacteria were in the range 0.4–1.6 mg/dm^3^, while biocidal concentrations were between 0.4–3.2 mg/dm^3^.

Karimipour and Tanomand [64] conducted research on the antibacterial effect of nanosilver particles against Gram-negative bacteria, i.e. *Pseudomonas Aeruginosa*. They applied suspension of nanoAg prepared by dispersing nanoparticulate powder in nutrient broth. The authors used two different types of nanoparticles. One of them consisted of nanoparticles of 20 nm and the other one of 5 nm nanoparticles. The concentration of nanoAg in the suspension varied by series dilution method, and it was equal to 312 to 40,000 mg/kg. The researchers performed tests by both well diffusion and disc diffusion method. In both techniques, the results were similar. One conclusion regards the influence of the nanoAg concentration and the other one concerns the influence of nanoparticles size. The higher the concentration of nanoAg, the stronger an anti-microbial effect may be achieved. The MIC values were equal to 75 mg/kg for 5-nm particles and 625 mg/kg for 20-nm particles. The MBC values were 156 mg/kg for 5-nm particles and 1250 mg/kg for 20-nm particles. Thus, the reduction of nanoparticle sizes leads to the achievement of more effective bacteriostatic and biocidal properties.

In the studies conducted by Krishnan et al. [65], it was confirmed that suspension of nanosilver dispersed in Brain Heart Infusion (BHI) broth at a concentration of 5 mg/ml is a biocidal factor in destroying 99.9% of *Enterococcus faecalis* strain. The size of nanoparticles was of 45–50 nm. Considering the fact that *Enterococcus faecalis* are highly resistant for the common agents, it may be concluded that nanosilver is able to manage with their pathogenic activity in a rewarding manner.

Very interesting studies were conducted by Rautela et al. [66]. Beside performing typical anti-microbial properties assessment, the researchers also tried to explain the possible mechanism of action of nanoAg against Gram-positive (*Bacillus cereus* and *Staphylococcus aureus*) and Gram-negative bacteria (*Escherichia coli*). It was evidenced that relatively low concentrations of green-synthesised silver nanoparticles were enough to achieve satisfying bacteriostatic effect. For *Bacillus cereus*, the concentration was equal to 5.2 mg/dm^3^, for *Staphylococcus aureus,* 2.6 mg/dm^3^ and for *Escherichia coli*, 2.0 mg/dm^3^. In order to assay the influence of nanoparticles on the content of extracellular matter after different times of contact, authors determined that the concentration of reducing sugars and proteins in supernatant obtained after centrifugation of suspension consists of nanoAg and bacteria inoculum. In each case, the content of both types of molecules was higher with time. It was concluded that silver nanoparticles may enhance the permeability of the cell membrane, which leads to the leakage of molecules necessary for life.

Also, anti-microbial properties of zinc oxide nanoparticles have been the object of the studies of other scientists. Yousef and Danial [67] conducted analysis on the activity of nanoZnO against food borne pathogens such as *Bacillus subtilus*, *Bacillus megaterium*, *Staphylococcus aureus*, *Sarcina lutea*, *Escherichia coli*, *Pseudomonas aeruginosa*, *Klebsiella pneumoniae*, *Proteus vulgaris*, *Candida albicans* and *Aspergillus niger*. While maintaining the concentration of nanoZnO in a dispersing agent at a constant level (20 mg/dm^3^), after preparation of agar cultures, the inhibiting zone was assessed. It has turned out that the greatest, not-grown areas were obtained in the case of *Bacillus subtilus*, *Escherichia coli* and *Staphylococcus aureus*. The worst inhibiting activity was achieved against *Aspergillus niger* and *Proteus vulgaris*. The authors also managed to determine the MIC and MBC values. The obtained results showed that the development of all studied strains was completely inhibited in the tested concentration range (0.5–20 mg/dm^3^). It has been concluded that zinc oxide nanoparticles may be an effective bacteriostatic against the development of the studied strains. On the other hand, their biocidal properties are not satisfying. However, authors claim that the price of zinc oxide is an encouraging factor in applying it in anti-microbial products. Iranian researchers [68] have documented the anti-microbial activity of zinc oxide nanoparticles against ten human pathogens, including *Escherichia coli, Klebsiella pneumoniae, Pseudomonas aeruginosa, Serratia marcescens, Salmonella typhi, Acinetobacter baumannii, Citrobacter freundii, Proteus mirabilis, Staphylococcus aureus* and *Bacillus cereus*. The bioassay was based on the agar well diffusion method. Both MIC and MBC values were also determined. For comparison, scientists used popular antibiotics (gentamicin, ampicillin, nalidixic acid, amoxicillin, amikacin, ciprofloxacin, co-trimoxazole, norfloxacin and cephalexin). The authors also observed concentration-dependence in destroying the strains. The inhibiting zone was greater when the content of nanoZnO was higher (from 2.5 to 160 mg/ml). Compared to the control group, which consisted of antibiotics, a significant bacteriostatic effect was noted. The group of strains that were most susceptible for inhibiting activity of zinc oxide nanoparticles included *S. aureus*, *S. marcescens* and *E. coli*. However, it was required to apply a higher concentration of nanoZnO to completely destroy these microorganisms (20 mg/ml). A lower concentration (10 mg/ml) was enough to kill *P. aeruginosa*, *A. baumannii, K. pneumoniae* and *S. aureus*.

The destroying action of ZnO nanoparticles against *Pseudomonas aeruginosa* has also been proved in the studies of Aysa and Salman [69]. Particles ranging from 23 to 29 nm served in the microbiological assessment. Some powder of nanoZnO were modified by addition of oleic acid. This operation ensured a better biocompatibility. It was figured out that a suspension at a concentration of 20 mg/dm^3^ was an effective inhibiting agent.

Also, Almoudi et al. [70] reviewed the studies on zinc oxide activity against *Streptococcus mutans*, which is common oral pathogen. Based on their research, it may be concluded that nanoZnO may play a role as an effective antibacterial agent, even at very low concentrations. The minimal inhibitory concentration is equal even to 0.39 mg/ml, and the minimal biocidal effect is achieved when applying nano ZnO at 3.125 mg/ml. Authors concluded that zinc oxide nanoparticles may play a role as active agents in both oral and dental preparations.

A very interesting application of silver combined with FeO_2_ and FO_2_ nanoparticles was presented in the work of Abdelhamid et al. [71]. Authors used such constructs for destroying different pathogenic microorganisms. Polyethylene glycol played a dual role. First at all it served as a stabilizing agent for nanoparticles. It also enhanced the biocompatibility to the live matter. It was found out that such products may be used as an efficient material acting as antibacterial agent. What is more, thanks to the fact that the nanoparticles have magnetic properties, they may be easily removed from the treated environment using a small magnetic bar. This feature makes the product highly usable.

Wu et al. [72] presented a novel method to destroy both Gram-negative and Gram-positive bacteria with using stannous dioxide (SnO_2_) modified with graphene (G) nanoparticles. The authors reported the activity against *Pseudomonas aeruginosa* and *Staphylococcus aureus* as model strains. Pure graphene nanoparticles were also tested. It was figured out that due to the synergic effect, merging SnO_2_ with graphene gives much better results in destroying both types of bacteria. Authors suggest that this result is achieved thanks to the fact that the applied nanoparticles block the gaps by which the nutrients are taken and that leads to the cell death.

Abdelhamid and Wu [73] conducted studies on using multifunctional graphene magnetic nanosheet decorated with chitosan as a biosensor for sensitive detection of pathogenic bacteria such as *Pseudomonas aeruginosa* and *Staphylococcus aureus*. This phenomena is possible thanks to the non-covalent interactions between the obtained construct pathogenic and bacteria. The detection of bacteria signals is achieved by a direct fluorescence measurements. Due to the fact that the product has increased ratio of surface area to its volume, its sensitivity is enhanced. Its relatively low price is also a great advantage.

## Conclusions

A series of PVA-based compositions with antibacterial nanoparticles has been prepared. The inhibiting properties of compositions with nanoZnO, nanoAg and nanoCu in development of both Gram-positive and Gram-negative bacteria have been confirmed. Antimicrobial activity was tested against *E. coli, P. aeruginosa* and *S. aureus* strains. Compositions with silver nanoparticles exhibited the best antibacterial effect against *E. coli* strain. Zinc oxide nanoparticles acted as the worst antibacterial agent in the case of Gram-negative bacteria. Copper nanoparticles gave the strongest inhibiting properties against *S. aureus* and *P. aeruginosa* strains. The structure of cell wall is a very important factor in assessment the antimicrobial properties. It influences on the migration of used agents. The size of applied nanoparticles is another crucial factor. In general, smaller nanoparticles give better antimicrobial effect. Thanks to the fact that it is possible to solidify the products, they may find applications in places in which the biocidal effect is particularly desired. The composition may be applied on a microbiologically contaminated surface, and after its solidification, it may be detached along with dead biocidal film. The content of some organic compounds, such as chitosan or casein, may be helpful in attracting the microbial film by forming the bonds between these molecules and the polymers that are excreted by the cells.

## Data Availability

All data generated or analysed during this study is available from corresponding author on reasonable request.
